# Supplementary material for: Polyphasic Systematics of the Fungicolous Genus Cladobotryum Based on Morphological, Molecular and Metabolomics Data
Source: J Fungi (Basel). 2022 Aug 20;8(8):877. doi: 10.3390/jof8080877 (PMC9409756; doi:10.3390/jof8080877)
Supplement: Supplementary file 1 [file jof-08-00877-s001.zip › jof-1865155-SM.pdf]

Journal of *Fungi*

Article

Polyphasic Systematics of the Fungicolous Genus *Cladobotryum* Based on Morphological, Molecular and Metabolomics Data

Nikola Milic [1,2] Anastasia C. Christinaki [3], Dimitra Benaki [4], Aimilia A. Stavrou [3,†], Nikolaos Tsafantakis [2], Nikolas Fokialakis [2,*], Vassili N. Kouvelis [3,*] and Zacharoula Gonou-Zagou [1,*]

Supplementary Materials

Text S1: Analytical Species Descriptions

Cladobotryum fungicola (G.R.W. Arnold) Rogerson & Samuels, Mycologia 85 (2): 262 (1993)

Strain examined: Greece: *Fthiotida:* Mesochori, Mt. Oiti, in *Quercus frainetto, Q. pubescens* and *Q. coccifera* forest with *Abies cephalonica* (sporadically), on basidiome of *Cortinarius* sp., 2008, coll./isol. *Z. Gonou-Zagou,* **ATHUM 6855.**

Mycelium: floccose to cottony; white, with prominent tufts; relatively symmetrically spreading, with irregular margin (Figure 1a). *KOH reaction:* negative. *Growth rate:* relatively high. *Colony reverse:* pale buff to yellow, progressively turning intensely yellow to bright orange (Figure 1b). *Conidiophores:* long, verticillate, branched mostly unilaterally; septate, at verticils the lower cells with characteristic swelling at their upper extremity; hyaline (Figure 1c). *Conidiogenous cells:* elongated, narrow-conical to lageniform, subulate, with very delicate, elongated, slightly wavy or zigzag-shaped apices; arranged in dense verticils, mostly apically, 1–10 per verticil; aseptate; hyaline (Figure 1c, d). *Conidia:* ellipsoidal to cylindrical or fusiform; (13)15–18(20) × 5–6.5 μm, Qm 2.8; mainly two-celled, rarely one-celled; sometimes slightly curved or swollen at base or intensely constricted at septa; hyaline, smooth; (Figure 2e – h). *Sclerotioid structures:* not observed. *Teleomorph:* not produced.

Comments: The micromorphological characters of our strain fit perfectly with the original description of the anamorphic state made by Arnold [11], except for the formation of sclerotioid aggregations, where the species is described as *Sibirina fungicola*. It can be distinguished by the numerous, delicate, elongated, verticillately arranged conidiogenous cells, along with the two-celled, sometimes curved conidia. The zigzag-shaped apices of conidiogenous cells, which seem to be quite characteristic, were vaguely illustrated by Põldmaa and Samuels [31], though they were not mentioned in the text, as well as in the original description. The teleomorph *Hypomyces semitransluscens* was first described by [12] and connected to the anamorphic species *Sibirina fungicola*, the latter been transferred to *Cladobotryum* [6]. In the aforesaid work, the conidia description in the text differs from those depicted in the corresponding illustration, the former fitting the original description, while the latter pointing to a different species. Although in several systematic works the species is referred by the teleomorphic name [9,23,31], in the on-line mycological databases MycoBank (https://www.mycobank.org) and Index Fungorum (http://www.indexfungorum.org) no such combination is mentioned and both species are treated separately.

Hosts of *Cladobotryum fungicola* recorded by now in the available literature belong to various genera of basidiomycetes, mainly aphyllophoraceous [22]. As far as our knowledge goes, this is the first record of the genus *Cortinarius* as its host.

Cladobotryum apiculatum (Tubaki) W. Gams & Hooz., Persoonia 6 (1): 97 (1970)

Strain examined: Greece: *Karditsa:* Ag. Nikolaos, Mt. Zigourolivado, in *Fagus sylvatica* forest, on basidiome of *Russula* sp., 2009, coll. *P. Delivorias,* isol. *A. Liakouri,* **ATHUM 6907**.

Mycelium: cottony, compactly to aerial; white, to off white; symmetrically spreading, with regular margin (Figure 2a). *KOH reaction:* rather positive. *Growth rate:* relatively high. *Colony reverse:* white, progressively turning off white, buff to ochre; sometimes visible brownish granules (Figure 2b). *Conidiophores:* long, verticillate, branched (especially in apical areas); septate; hyaline (Figure 2c). *Conidiogenous cells:* elongated, cylindrical, subulate, with obtuse apices; in verticils, 2-4 per verticil; aseptate; hyaline (Figure 2c, d). *Conidia*: ovoid, ellipsoidal to broadly cylindrical or clavate; (11)13–28(33) × (3)5–9.5(11) μm, Qm 3; one-celled, very rarely two-celled; quite often swollen at base; hyaline, smooth; many easily germinating from both edges (Figure 2e, f). *Sclerotioid structures:* multicellular, appearing as chains of (sub)globose cells, constricted at septa, sometimes branched; thick-walled, smooth; sometimes forming small aggregations on the medium surface (Figure 2g – h). *Teleomorph:* not produced.

Cladobotryum verticillatum (Link) S. Hughes, Canadian Journal of Botany 36 (6): 750 (1958)

Strains examined: Greece: *Arkadia:* Megalopoli, Mt. Lykaio, in *Quercus* sp. forest, on basidiome of *Lactarius subumbonatus,* 2009, coll. *M. Triantafyllou,* isol. *Z. Gonou-Zagou,* **ATHUM 6850**; Megalopoli, Mt. Lykaio, in *Quercus* sp. forest, on basidiome of *Lactarius subumbonatus,* 2010, coll. *M. Triantafyllou,* isol. *A. Liakouri* **ATHUM 6920**; Megalopoli, Mt. Lykaio, in *Quercus* sp. forest, on basidiome of *Lactarius subumbonatus,* 2010, coll. *M. Triantafyllou,* isol. *A. Liakouri* **ATHUM 6921.**

Mycelium: felt-like to cottony; white to off white; symmetrically spreading, with regular margin (Figure 3a). *KOH reaction:* negative. *Growth rate:* relatively high. *Colony reverse:* white, progressively turning off white to very pale buff (Figure 3b). *Conidiophores:* long, verticillate, branched (intensely in apical areas); septate; hyaline (Figure 3c). *Conidiogenous cells:* elongated, cylindrical, subulate, with obtuse apices; arranged in verticils, 1–4(5) per verticil; aseptate, rarely 1-septate; hyaline (Figure 3c). *Conidia:* subglobose to ovoid or broadly ellipsoidal; 11–24(27) × (6)8–11(13) μm, Qm 2.2; one-celled; extremely rarely two-celled; hyaline, smooth; many easily germinating from both edges; few joined at their base or laterally (Figure 3d – f). *Sclerotioid structures:* multicellular, appearing as chains of (sub)globose, ellipsoidal to cylindrical cells, constricted at septa; thick-walled, smooth or rarely slightly rough; formed apically or intercalarily on hyphae; sometimes clustered in aggregations (Figure 3g, h). *Teleomorph:* not produced.

Comments: Our strains of *C. apiculatum* and *C. verticillatum* are quite similar macromorphologically in culture. The colonies are white, both almost retaining their white colour during maturity, while on the reverse side exhibiting pale pigmentation. In terms of micromorphology, the most notable difference between the two species is the presence of mainly one-celled, mostly subglobose to ovoid conidia in *C. verticillatum,* in contrast to the one- to rarely two-celled, ovoid to broadly elongated conidia in *C. apiculatum*. Considering the morphology of the sclerotioid structures, in both species the cells are smooth-walled, rarely rough-walled in *C. verticillatum*, a feature opposing to the existing references where the two species are distinguished by the roughened formations in *C. apiculatum* in contrast to the smooth-walled ones in *C. verticillatum* [29]. It is worth mentioning that *C. apiculatum* may form screrotial aggregations on the medium surface that are only visible from the underside.

There is a correlation made between *C. verticillatum* and *Hypomyces armeniacus* in culture [30,101], but not according to MycoBank (https://www.mycobank.org) and Index

Fungorum (http://www.indexfungorum.org), both of which acknowledge the two species as distinct and *C. verticillatum* as unliked to any teleomorph. Nevertheless, a molecular phylogenetic study suggests *C. verticillatum* is linked to *H. armeniacus* [46].

Both species have a preference to russuloid basidiomes. *Cladobotryum apiculatum* occurs on *Russula* spp. as expected [22]. All *Cladobotryum verticillatum* strains were isolated from the basidiomycete *Lactarius subumbonatus,* collected from the same locality, but in different time of year. *Cladobotryum verticillatum* seems to grow strictly on the hosts belonging to the russuloid genera *Lactarius* and *Russula* [22] . However, there are some references that allude to boleticolous [8,30,102] nature of *Cladobotryum verticillatum,* as well as records of this species growing on true agarics (Agaricales) [103]. To the best of our knowledge, this is the first report of *Lactarius subumbonatus* as a host of the genus *Cladobotryum*.

Cladobotryum varium Nees, System der Pilze und Schwämme: 56, t. 4:54 (1817)

Strains examined: Greece: *Attiki:* Mt. Parnitha, in *Abies cephalonica* forest, on basidiome of *Clitocybula familia*, 2008, coll./isol. *Z. Gonou-Zagou,* **ATHUM 6845**; Mt. Parnitha, in *Abies cephalonica* forest, on basidiome of *Cortinarius* sp., 2009, coll./isol. *Z. Gonou-Zagou,* **ATHUM 6856**; Mt. Parnitha, in *Abies cephalonica* forest, on basidiome of *Panellus* sp., 2009, coll./isol. *Z. Gonou-Zagou,* **ATHUM 6846**; *Eurytania:* near Krikello, in *Abies boresii-regis* forest, on basidiome of *Inocybe* sp., 1998, coll./isol. *Z. Gonou-Zagou,* **ATHUM 6514**; near Krikello, in *Abies borisii-regis* forest, on polypore basidiome, 1998, coll./isol. *Z. Gonou-Zagou,* **ATHUM 8003**; Mt. Tymfristos, in *Abies borisii-regis* forest with *Juniperus oxycedrus* (sporadically), on basidiome of *Ganoderma* sp., 2004, coll./isol. *Z. Gonou-Zagou,* **ATHUM 8002**; *Karditsa:* Ag. Nikolaos, Mt. Zigourolivado, in *Fagus sylvatica* forest, on agaricoid basidiome, 2009, coll. *P. Delivorias,* isol. *A. Liakouri* **ATHUM 6908**; *Rodopi:* Frakto virgin forest on polypore basidiome, 2000, coll. *Th. Angelopoulos,* isol. *Z. Gonou-Zagou* **ATHUM 7996**; *No data:* on polypore hymenophore, 1997, coll./isol. *Z. Gonou-Zagou,* **ATHUM 7995**.

Mycelium: slightly felt-like or cottony to powdery or with floccose to powdery tufts radially or irregularly arranged; white, sometimes yellow at centre; usually when mature with bright yellow-orange irregular patches; symmetrically spreading, with even margin (Figure 4a). *KOH reaction:* strongly positive/red. *Growth rate:* relatively high. *Colony reverse:* buff, progressing towards various yellow hues with ochre and orange undertones (Figure 4b). *Conidiophores:* long, verticillate, branched (predominantly in apical areas); septate; hyaline (Figure 4c – f). *Conidiogenous cells:* heterogeneous in shape, elongated, cylindrical, truncated-conical to lageniform, subulate, with more or less obtuse apices; arranged in verticils, 1–5(6) per verticil, emerging almost parallelly to conidiophore axis; some progressively reduced in length; aseptate; hyaline (Figure 4c – f). *Conidiogenesis:* blastic; retrogressive production of conidia (Figure 4c – f). *Conidia:* ellipsoidal to shorty cylindrical; (10)13–17(20) × (5)6–8(9.5) μm, Qm 2.1; mainly two-celled, rarely one-celled or three-celled; sometimes constricted at septa; base with small apiculus or truncate, sometimes with a ring-like formation; many produced in a slanting manner; hyaline, smooth; predominantly single, sometimes forming chains, often branched (Figure 4g, h). *Sclerotioid structures:* multicellular, appearing as chains of (sub)globose, oblong to ellipsoidal cells, constricted at septa; thick-walled, smooth; formed apically on hyphae (Figure 4i). *Teleomorph:* not produced.

Comments: Our isolates exhibit inter- and intra-strain variability. Nevertheless, the colonies of *Cladobotryum varium* are distinguished macromorphologically from those of all other studied species by their remaining white, powdery mycelium, the irregular orange patches, usually formed on their surface, and the buff-yellow-orange reverse. Microscopically, the conidia are mostly ellipsoidal, two-celled and in the strains ATHUM 6846, 4856 forming characteristic long chains, some of which branched. This uncommon

feature was also observed by Matsushima [81] in only one strain (MFC 1816=CBS 675.77) and referred to by Rogerson & Samuels [6]. Conidial connections via anastomoses in a strain of *C. varium* were also observed by Arnold & Yurchenko [82]. Moreover, it is quite typical for the conidiogenous cells to be arranged verticillately and almost in a parallel manner to the main axis of a conidiophore, especially in the apical areas of the conidiogenous apparatus. This feature is mostly seen in microphotographs accompanying taxonomic descriptions but is unaddressed in the main text [6,82]. In addition, the heterogeneity of conidiogenous cells is apparent in our strains, especially in certain colonies of ATHUM 8002 and ATHUM 6908, where the cells become gradually shorter, from their truncated apices two types of conidia are gradually produced—ellipsoidal conidia truncated at base and cylindrical conidia with both sides truncated—revealing a probable retrogressive mode of conidiogenesis. In any case, the conidiogenesis is very characteristic and similar only to that of *C. mycophilum*.

H. aurantius is referred as the teleomorph of *C. varium* based on strains containing both states [6,8,9,82], a correlation not referred or accepted by either MycoBank (https://www.mycobank.org) and Index Fungorum (http://www.indexfungorum.org), since they treat both species separately.

The hosts of *Cladobotryum varium* belong to various basidiomycete groups (Agaricales, aphyllophorales and rarely heterobasidiomycetes) [22] . However, the basidiomycetes *Clitocybula familia, Ganoderma* sp., *Inocybe* sp. and *Panellus* sp., from which our strains were isolated, as far as our knowledge goes, are not reported as hosts of *Cladobotryum varium* so far. *Ganoderma oregonense* is reported as a host of *Hypomyces aurantius* [22] .

Cladobotryum mycophilum (Oudem.) W. Gams & Hooz., Persoonia 6 (1): 102 (1970)

Strains examined: Greece: Attiki: cultivated *Agaricus bisporus,* 2010, coll./isol. *Z. Gonou-Zagou,* **ATHUM 8001**. *Eurytania:* Ag. Nikolaos, in *Platanus orientalis* forest with *Quercus* sp., *Abies cephalonica* and *Castanea sativa* (sporadically), on basidiome of *Mycena* sp., 2010, coll./isol. *Z. Gonou-Zagou,* **ATHUM 8000**; *Karditsa:* Belakomitis, in *Abies borisii-regis* forest, on basidiome of *Inocybe* sp., 1999, coll./isol. *Z. Gonou-Zagou,* **ATHUM 7994.** *Magnisia:* Mt. Pilio, in *Castanea* sp. forest, on basidiome of *Hypholoma* sp., 2009, coll. *P. Delivorias,* isol. *A. Liakouri* **ATHUM 6906**.

Mycelium: felt-like at first, becoming fibrous, floccose to cottony; white, yellow, buff to ochre, progressing towards various red hues with brownish and purplish undertones; when mature with white powdery tufts, aerial or superficial, irregularly or radially arranged; symmetrically or asymmetrically spreading, with regular margin; sometimes distinct, minute brownish granular formations; rarely production of perithecia (Figure 5a). *KOH reaction:* negative. *Growth rate:* relatively high. *Colony reverse*: yellow, buff, progressing towards ochre, pinkish hues, and various red hues with brownish undertones (Figure 5b). *Conidiophores*: long, verticillate, branched (intensely in apical areas); septate; hyaline (Figure 5c – e). *Conidiogenous cells*: phialides, heterogeneous in shape, elongated to cylindrical, subulate, sometimes lageniform, pyriform to ampulliform; when mature somewhat conical with obtuse or truncate apices; arranged in verticils, 1–5(6) per verticil, mainly 3–4; aseptate, some septate in maturity; hyaline (Figure 5c – f). *Conidiogenesis*: blastic; retrogressive production of conidia (Figure 5c – f). *Conidia*: ovoid to broadly ellipsoidal to ellipsoidal or cylindrical; mainly 2-celled, many 3-celled, few 4-celled and rarely one-celled; some curved; variably sized, 2-celled (16)20–28(32) × (7)9–11.5(13), Qm′ 2.1, 3-4-celled 25–35(39) × 9–12 μm, Qm′′ 3.1, hyaline, smooth; at base with apiculi sometimes indistinct or truncate; characteristic production in a slanting manner indicative of retrogressive conidiogenesis; mature conidia with hyaline pore-like protrusions, some joined in twos; production of secondary conidia on a conidiogenous cell emerging directly

from a conidium; (Figure 5g – k). *Sclerotioid structures:* multicellular, as chains of globose, ellipsoidal to elongated cells or irregular aggregations of variably shaped cells; cells thick-walled, smooth, seldom slightly rough (Figure 5l). ***Teleomorph:*** only in strain ATHUM 8001; *Perithecia:* mostly subglobose to pyriform, reddish to purplish brown; on ostiole either slightly pigmented droplets or light yellowish mass of ascospores; partially immersed in the subiculum (Figure 5m). *Ascospores:* fusiform to oblong, apiculate; 25–30(32) × 4–5.5(6.5) μm (incl. apiculi); 2-celled, sometimes with constriction at median septum; many curved; apiculi rather conical, acute to rarely obtuse; 4.5–5.5(6.5) μm; slightly verrucose (Figure 5n).

Comments: All the strains studied exhibited intra- and inter-strain variability as far as macro- and micro-morphology is concerned—i.e., colony texture (including formations such as asexual sporulation, sclerotioid aggregations, sexual reproduction), colour and pigment production, and the corresponding microscopic characters. Nevertheless, our descriptions—and the flunctuations of the characters—fit perfectly with the protologue of *Hypomyces odoratus* [10], where both sexual and asexual reproduction were described from culture. The connection of the anamorphic genus *Cladobotryum mycophilum* with the teleomorphic *H. odoratus* is referred to in several publications [8–10,29,35,36], though both species are treated as separate in the on-line databases MycoBank (https://www.mycobank.org) and Index Fungorum (http://www.indexfungorum.org). According to our observations, the anamorph is characterised by mainly two-celled conidia, but also 3–4-celled, varying in size and shape, produced retrogressively from gradually shortening conidiogenous cells. Conidiogenesis in *C. mycophilum* is *similar* to that of *C. varium* and very characteristic of the retrogressive way of production. It is worth noting that secondary conidia seem to be produced from mature conidia and most probably the hyaline protrusions of the latter indicate the place of their blastic formation. Joined conidia were depicted as a drawing by Arnold [10], though this trait was not mentioned in the text. Sclerotial aggregations were formed, either microscopic or macroscopic. Sexual reproduction was observed in the strain ATHUM 8001 isolated from a cultivated basidioma of *Agaricus bisporus*. Perithecia were formed in the very first cultures, with the strain losing this ability with time. The teleomorphs seem to be produced only in culture and are described very few times [8–10,46], while the fungus appears as anamorph in nature.

The hosts of *Cladobotryum mycophilum* belong to various genera of macrofungi, mainly agarics [22]. Species of the genus *Hypholoma*, from which one of the studied strains was isolated, to the best of our knowledge is not hitherto recorded as a host of *C. mycophilum*.

Hypomyces rosellus (Alb. & Schwein.) Tul. & C. Tul., Annales des Sciences Naturelles Botanique 13: 12 (1860)

Anamorph: ***Cladobotryum dendroides*** (Bull.) W. Gams & Hooz., Persoonia 6 (1): 103 (1970)

Strains examined: Greece: *Attiki:* Athens "National Gardens", on basidiome of *Flammulina velutipes*, 2008, coll./isol. *Z. Gonou-Zagou,* **ATHUM 6847**; Mt. Parnitha, in *Abies cephalonica* forest, on basidiome of *Tricholoma* sp., 2008, coll./isol. *Z. Gonou-Zagou* **ATHUM 6849**; Mt. Parnitha, in *Abies cephalonica* forest, on basidiome of *Hohenbuehelia* sp., 2000, coll./isol. *Z. Gonou-Zagou* **ATHUM 7998**. *Eurytania:* Ag. Nikolaos, in *Platanus orientalis* for with *Quercus* sp., *Abies cephalonica* and *Castanea sativa* (sporadically), on ascocarp of *Helvella lacunosa,* 2010, coll./isol. *Z. Gonou-Zagou,* **ATHUM 7999**. *Karditsa:* Ag. Nikolaos, Mt. Zigourolivado, in *Fagus sylvatica* forest, on polypore basidiome, 2009, coll. *P. Delivorias,* isol. *A. Liakouri* **ATHUM 6909**. *Xanthi:* Mt. Leivaditis, in *Fagus sylvatica* forest

with *Juniperus communis,* on basidiome of *Polyporus varius,* 2009, coll. *A. Sergentani,* isol. *Z. Gonou-Zagou,* **ATHUM 6848**.

Mycelium: felt-like to floccose, somewhat powdery, or with somewhat radially arranged floccose-powdery tufts; whitish, buff, progressing towards ochre, pinkish hues, and various red hues with purplish and brownish undertones; symmetrically spreading, with regular margin (Figure 6a). *Colony reverse:* buff to ochre, progressing towards ochre and various red hues with brownish and purplish undertones (Figure 6b). *KOH reaction:* strongly positive/red-violet. *Growth rate:* relatively high. *Conidiophores:* long, verticillate, branched (intensely in apical areas); septate; hyaline (Figure 6c – e). *Conidiogenous cells:* elongated, cylindrical, subulate, with apparent wavy/zig-zag-shaped apices; arranged in verticils, 1–6 per verticil, mainly 3–5, often 1–3 at branching points; aseptate; hyaline (Figure 6c – e). *Conidiogenesis:* polyblastic; multiple conidiogenous loci per conidiogenous cell with sympodially emerging conidia; rarely, a secondary sympodial development observed, as a branching of an already existing (Figure 6d – g). *Conidia:* ovoid, broadly ellipsoidal to mainly ellipsoidal or cylindrical; (16)21.5–28(35) × (8.5)10–11.5(13) µm, Qm 2.3; two-, three- or four-celled, with prominent apiculus; hyaline, smooth; few slightly curved; mature conidia sometimes constricted at septa; some joined in twos at base or forming short chains (Figure 6h – k). *Sclerotioid structures*: not observed. *Teleomorph:* not produced.

Comments: Progressive pigmentation of both the colony and substrate from ochre into red hues is one of the main macromorphological traits of all red-pigmented, presumably aurofusarin-producing, phylogenetically related species of the genus *Cladobotryum*, including *Cladobotryum dendroides* [2,3,7,35,36]. Still, sympodial conidiogenesis is the main diagnostic micromorphological character of all strains of *C. dendroides*, setting it apart from other *Cladobotryum* species. In addition, the long, 3-4 celled conidia contribute to the distinction of the species.

The teleomorphic *Hypomyces rosellus* is referred to by both MycoBank (https://www.mycobank.org) and Index Fungorum (http://www.indexfungorum.org) as the current name of the anamorphic *Cladobotryum dendroides.*

The hosts of *Cladobotryum dendroides* belong to various genera of Russulales, Agaricales and the aphylloporoid basidiomycetes [22]

To the best of our knowledge, the hosts of the Greek speciemens of *C. dendroides*, belonging to the basidiomycetous species *Flammulina velutipes* and *Hohenbuehelia* sp., as well as to the ascomycetous *Helvella lacunosa*, are not recorded so far as hosts of *C. dendroides*.

Cladobotryum rubrobrunnescens Helfer, Libri Botanici 1: 55 (1991)

Specimen examined: Germany, on *Inocybe* sp., 1989, *A. Resinger*, **CBS 176.92, ex-type strain**.

Mycelium: felt-like to compact cottony; whitish, yellow, buff, progressing towards ochre and various red hues with pinkish and purplish undertones; when mature copper coloured, either in scattered patches or at margin; symmetrically spreading, with regular margin (Figure 7a). *Growth rate:* relatively high. *KOH reaction:* positive. *Colony reverse:* buff, progressing towards ochre and various red hues with pinkish, purplish, or brownish undertones (Figure 7b). *Conidiophores:* very long, delicate, (sub)verticillate, unbranched, or sparsely branched unilaterally or alternately; septate; hyaline (Figure 7c, d). *Conidiogenous cells:* elongated, delicate, narrow-cylindrical, with obtuse apices; arranged in verticils, sometimes emerging almost orthogonally to conidiophore axis, often dichotomously at conidiophore apices, 1–4(5) per verticil, often 2–3 at the apex; aseptate; hyaline (Figure 7c, d). *Conidiogenesis:* blastic; a single or more conidiogenous loci per

conidiogenous cell and a single conidium or up to 6 conidia in a wreath-shaped arrangement (Figure 7c, d). *Conidia:* ellipsoidal or fusiform to cylindrical to bacilliform, rarely constricted at septa; in slide culture (21)22–26 (29) × (3.5)4–5 (5.5) µm, Qm 5.4, in petri dish culture (14)17–26(29) × (5.5)6–8.5(9.5) µm, Qm 2.9; mainly two-celled, sometimes the basal cell swollen, also one-celled or rarely three- or four-celled; hyaline, smooth; sometimes mature conidia joined in twos (Figure 7e – j). *Sclerotioid structures:* not observed. *Teleomorph*: not produced

Cladobotryum tenue Helfer, Libri Botanici 1: 57 (1991)

Specimen examined: Germany: *Regesburg-Keilberg*, on agaric., 1986, *H. Besl*, **CBS 152.92, ex-type strain**.

Mycelium: floccose to sparsely cottony; whitish, yellow, buff, progressing towards pale ochre to pinkish, rarely to various red hues; symmetrically spreading, with regular margin (Figure 8a). *KOH reaction:* negative. *Growth rate:* relatively high. *Colony reverse:* buff, progressively turning from ochre to bright terracotta and various red hues with pinkish, purplish to brownish undertones (Figure 8b). *Conidiophores:* very long, delicate, unbranched, or very sparsely branched, unilaterally or dichotomously at conidiophore apices, or solitary, shorter, emerging orthogonally from very long hyphae; septate; hyaline (Figure 8c – e). *Conidiogenous cells:* elongated, delicate, narrow-cylindrical to almost filiform, subulate, with obtuse apices; arranged in verticils at conidiophore apices, sometimes emerging almost orthogonally to conidiophore axis, often dichotomously at conidiophore apices, 1–3 per verticil, mainly 2 at the apex; aseptate; hyaline (Figure 8c – e). *Conidiogenesis:* blastic; a single or more conidiogenous loci per conidiogenous cell and a single conidium or up to 2 conidia in a V-shaped arrangement (Figure 8c – e). *Conidia:* ellipsoidal to clavate, cylindrical to bacilliform, rarely constricted at septa; in slide culture (21)22 – 28.5(36) × (3.5)4.5 – 5.5(6) µm, Qm 5.5, in petri dish culture 23–34.5(38) × 4–6, Qm 6.4; mainly two-celled, rarely one- or three-celled, hyaline, smooth; (Figure 8f – l). *Sclerotioid structures:* multicellular, elongated, appearing as chains of (sub)globose and pyriform cells, constricted at septa; thick-walled, smooth; formed intercalarily on hyphae (Figure 8m). *Teleomorph*: not produced.

Comments: There are only the protologue descriptions of *C. rubrobrunnescens* and *C. tenue* ever made so far [7]. Both species are grouped together with other red-pigmented, presumably aurofusarin-producing, species of the genus *Cladobotryum* due to the reddish exudates produced [2,7,35,36]. In terms of macro- and micro-morphology, our observations on PDA culture generally comply with the original descriptions, where the medium malt peptone agar (MPA) was used. Some fluctuations in spore shapes and sizes can be attributed to the species variability due to the different culture medium and conditions used. In slide culture of *C. rubrobrunnescens* the conidia are distinctively much narrower. As main diagnostic microscopic features of the two species in petri dish culture, the longer and narrower conidia can be considered, as well as the simpler conidiophores of *C. tenue.*

The ecology of *Cladobotryum rubrobrunnescens* is insufficiently studied, with only a single host reported, belonging to the agaricoid genus *Inocybe*. Besides the unidentified agaricoid host reported in the original description of *C. tenue,* there are subsequent reports from *Russula* sp., *Lactarius* sp. and *Tricholoma* spp. [35].

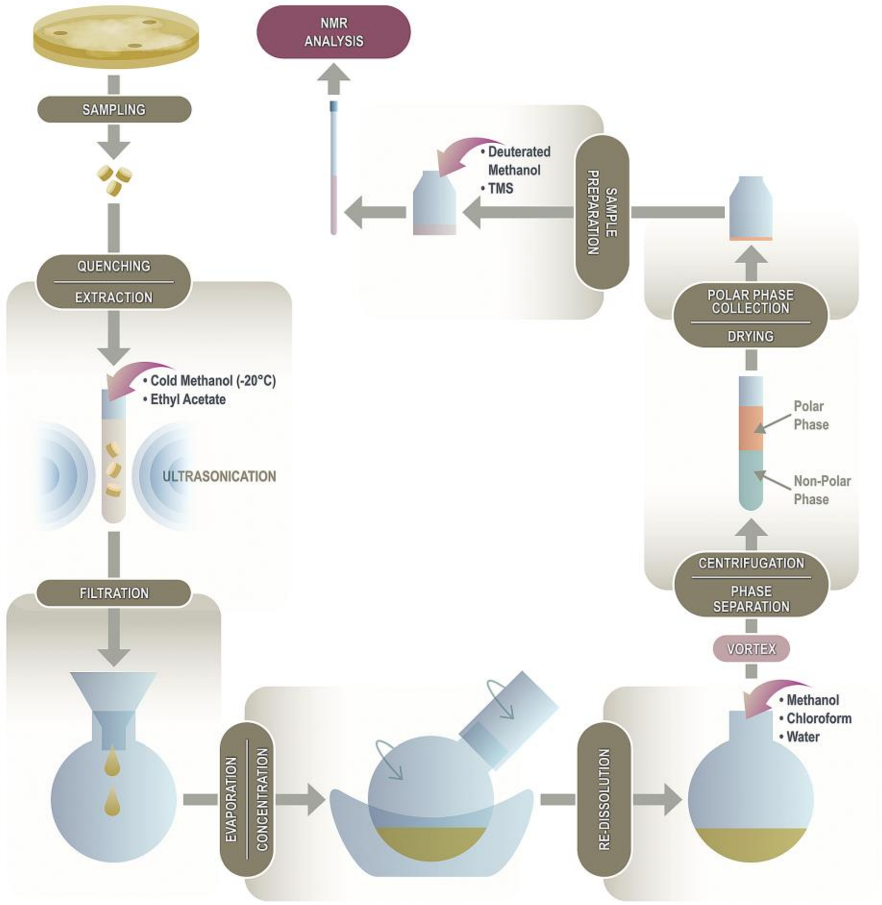

Figure S1. Simplified diagram of the extraction protocol followed in the present study.

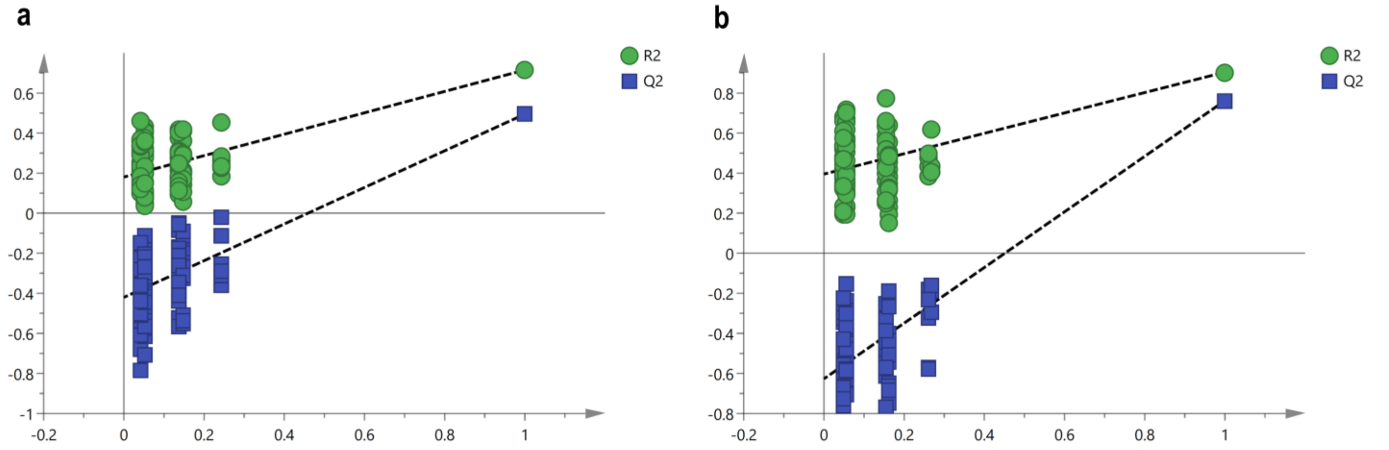

Figure S2. Permutation tests, applying 100 random changes, of the PLS-DA models of (**a**) all the examined *Cladobotryum* species presented in Figure 18b and (**b**) the red-pigmented species presented in Figure 19b.

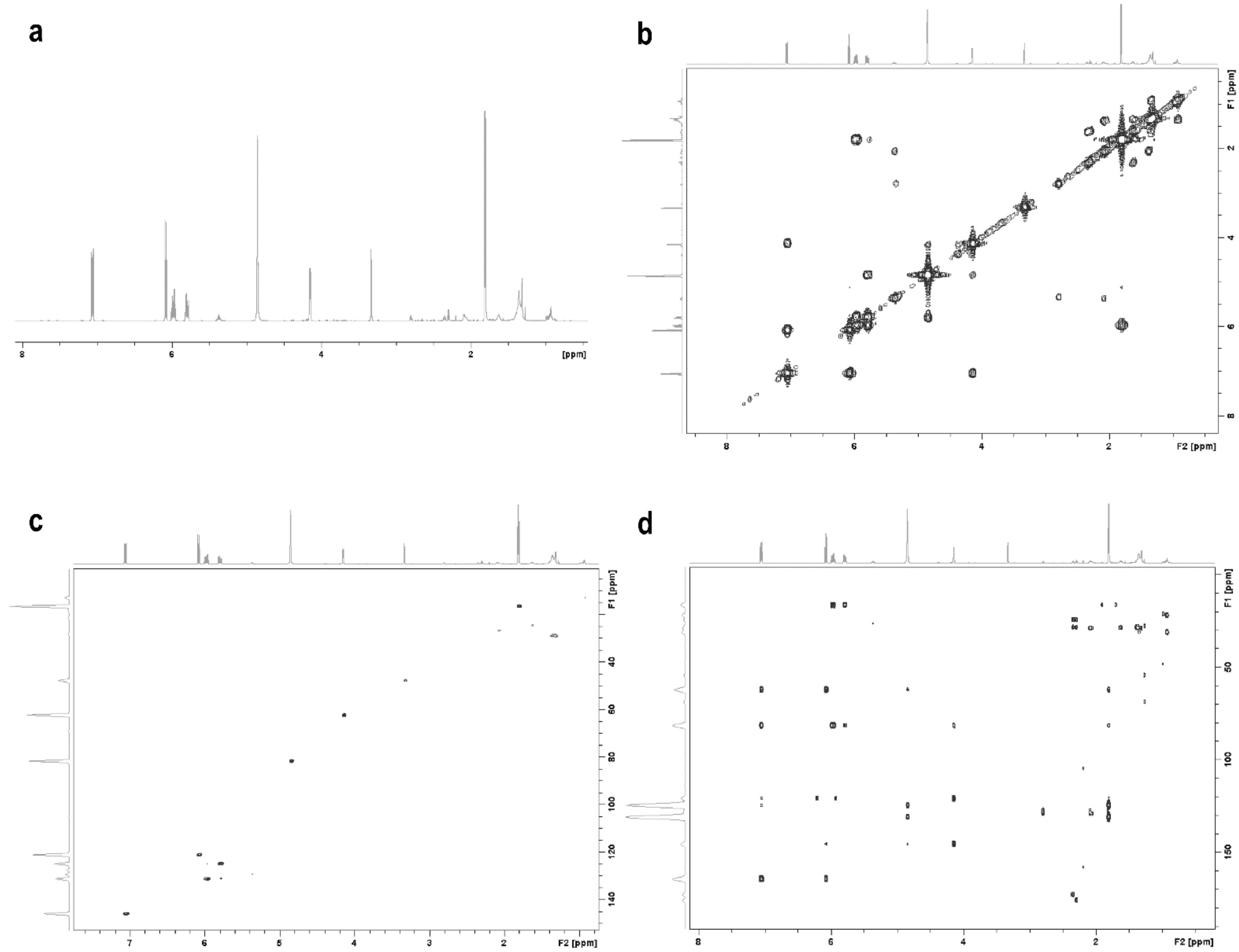

Figure S3. (**a**) 1H NMR, (**b**) COSY, (**c**) HSQC, (**d**) HMBC spectra of phomalactone in MeOD.
